# Fractured neck of femur below long spinopelvic fixation for Charcot spine: a case report

**DOI:** 10.1186/1752-1947-7-277

**Published:** 2013-12-30

**Authors:** Gerald MY Quan, Peter Wilde

**Affiliations:** 1 Department of Spinal Surgery, The Austin Hospital Melbourne, University of Melbourne, 8th Floor Harold Stokes Building, PO Box 5555, Heidelberg, VIC 3084, Australia; 2 Department of Orthopaedics, The Austin Hospital Melbourne, University of Melbourne, 8th Floor Harold Stokes Building, PO Box 5555, Heidelberg, VIC 3084, Australia

## Abstract

**Introduction:**

We present a case of a patient with a previously undescribed complication: intertrochanteric femoral neck insufficiency fracture after long-segment instrumented spinopelvic fusion to the ilium for Charcot spine.

**Case presentation:**

A 42-year-old Caucasian man with post-traumatic complete T6 paraplegia presented to our institution after developing Charcot spinal arthropathy at L3 and L4 and symptoms of autonomic dysreflexia 21 years after his original spinal cord injury. Multiple anterior and posterior surgeries were required to eventually achieve stabilization of his thoracolumbar spine to his pelvis and resolution of symptoms. The most distal fixation point was two iliac wing screws bilaterally. At 10 weeks after the final spinal surgery and after posterior spinal bony consolidation had occurred, he sustained an intertrochanteric femoral neck fracture, distal to the iliac fixation, whilst bending forward in his wheelchair. His proximal femoral fracture was internally fixed with an intramedullary device.

**Conclusions:**

Spinal Charcot’s arthropathy is a rare condition that may occur in patients with post-traumatic spinal cord injury. Although associated with high risk of complications, circumferential instrumented fusion in Charcot spine can restore spinal stability. Insufficiency fractures of the proximal femur are possible complications of long spinopelvic fusions.

## Introduction

After Charcot described the neuropathic joint in 1868 [1] and Charcot spinal arthropathy was reported in a patient with tabes dorsalis in 1884 [2], the first report of Charcot spinal arthropathy in a patient with post-traumatic paraplegia appeared in 1978 [3]. Since then, traumatic spinal cord injury has become the commonest cause of Charcot spinal arthropathy, however, this condition is rare with less than 90 cases reported [4]. The mean time of development of Charcot spine after the initial spinal injury is usually greater than 10 years [5]. The destructive process is triggered by loss of protective pain and proprioceptive sensation in the post-traumatic spinal cord-injured spine, resulting in abnormal motion and instability, leading to disc degeneration, facet joint destruction, and eventually causing vertebral destruction and fracture, subluxation, deformity, and dislocation. Symptoms include pain, further neurological deterioration, progressive deformity and impairment of sitting balance. There have been rare case reports of patients presenting with autonomic dysreflexia, an imbalance in reflex sympathetic discharge resulting in hypertension, headache and profuse sweating [4,6]. Surgery is often indicated upon failure of non-operative management, although it is associated with high risk of complications, including infection, failure of fixation and need for revision surgery [7,8]. The goals of surgery are to achieve a painless well-balanced spine, maintain sitting balance and obtain a solid fusion, thus restoring stability. Dual anterior and posterior surgery is usually recommended to obtain circumferential stabilization and fusion, especially in severe cases [7,8]. Occasionally, patients may have a long pre-existing fusion mass with the Charcot arthropathy distal to this, and the posterior instrumentation needs to be extended to the sacrum or pelvis [5].

Long multisegmental posterior thoracolumbar fusions to the sacrum and pelvis are challenging operations associated with high complication rates including instrumentation prominence and failure and pseudarthrosis [9]. However, successful fusion results in stiffening of the spine and leads to increased motion and biomechanical strain at caudal or rostral levels of the axial skeleton adjacent to the fusion [10]. This in turn may cause degeneration, instability and insufficiency fracture, especially in patients with poor bone quality and longer fusion segments [11]. Although uncommon, sacral insufficiency fractures just distal to S1 screws after multisegmental lumbosacral fusion have been reported in several case series [12]. If symptoms do not improve with non-operative management, extension of instrumentation to the iliac wings is an effective salvage procedure. However, the biomechanical effects of long fusions and instrumentation to the ilium are unknown. To the best of our knowledge, no previous case of bony fracture immediately distal to iliac fixation has been reported. When the fusion ends in the ilium, the hip joint becomes the adjacent distal joint. We present a patient who sustained a comminuted intertrochanteric femoral neck fracture after a long multisegment spinopelvic fixation to the ilium for Charcot spine.

## Case presentation

**Figure 1:**
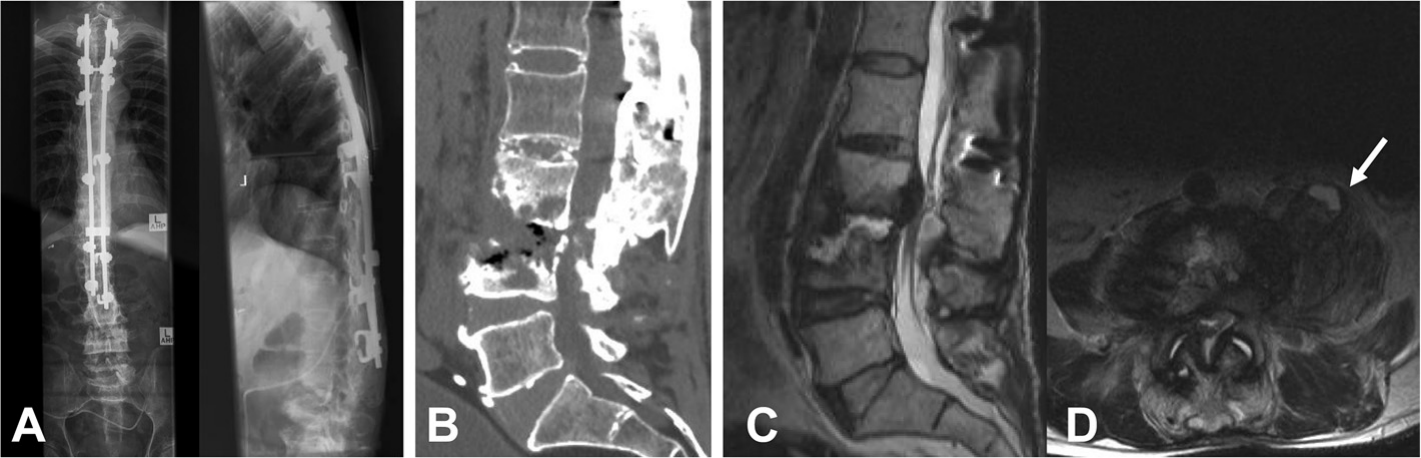
**Plain radiographs, computed tomography and magnetic resonance imaging showing bone destruction and deformity consistent with Charcot spine below previous thoracolumbar instrumented fusion. (A)** Plain anteroposterior and lateral radiographs showing previous instrumented fusion with hooks and rods from T3 to L3. Bony destruction and deformity is evident distal to the previous fusion. Sagittal computed tomography scan **(B)** and T2-weighted magnetic resonance imaging **(C)** showing extensive endplate and osseous destruction at L3 and L4. **(D)** Axial T2-weighted magnetic resonance imaging showing abnormal paravertebral soft tissue and a complex paraspinal mass within the psoas muscle with fluid collections, areas of dystrophic ossification and nonspecific soft tissue inflammatory changes (arrow).

A 42-year-old Caucasian man had a 21-year history of a traumatic spinal cord injury and complete T6 paraplegia due to a motorcycle accident in 1989, which was initially treated non-operatively. He subsequently developed a syrinx from C3 to T5 associated with post-traumatic deformity and underwent posterior instrumentation with hooks, rods and posterolateral fusion from T3 to L3 in 1994 (Figure 1). In 2005 he sustained bilateral distal femoral shaft fractures after falling from his wheelchair and underwent bilateral retrograde femoral intramedullary nail fixation which achieved successful union of the fractures. He presented with a 6-month history of lethargy, increasing lower torso and lower limb spasms associated with severe sweating, hypertension and recurrent headaches. He noticed a change in his posture, with kyphotic deformity in his lumbar spine and was no longer able to sit upright in his wheelchair. He also developed symptoms of urinary retention whereas previously he could initiate urinary voiding with bladder tap.

Plain X-rays showed severe bony reabsorption and destruction distal to the previous fusion coupled with new bone formation around the destroyed L4 vertebra (Figure 1). A computed tomography (CT) scan showed extensive endplate and osseous destruction of his L3 and L4 vertebral bodies, with gas vacuum phenomenon at this level. A magnetic resonance imaging scan confirmed the bony and soft tissue destruction at L3 and L4, as well as showing abnormal paravertebral soft tissue and a complex paraspinal 7cm diameter mass within his psoas muscle with associated multifocal and multilocular fluid collections, areas of dystrophic ossification and nonspecific soft tissue inflammatory changes.

**Figure 2:**
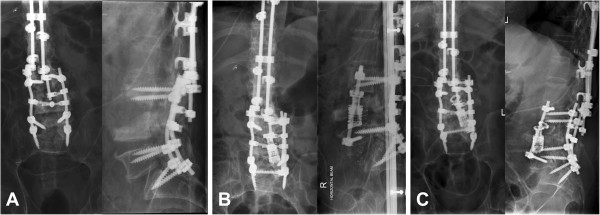
**Plain radiographs following the first and second surgeries showing posterior extension of instrumentation to the sacrum with pedicle screws and rods, anterior reconstruction with an expandable titanium cage and anterior rod and screws, and subsequent disengagement of the cage and shifting of the posterior instrumentation. (A)** Anteroposterior and lateral radiographs taken after the first surgery showing posterior extension of instrumentation from L3 to S1 with pedicle screws and rods. **(B)** Radiographs taken immediately after the second surgery involving L4 corpectomy and reconstruction with an expandable titanium cage and anterior rod and screws. **(C)** Radiographs taken after the patient was allowed to mobilize, showing disengagement of the cage and shifting of the posterior instrumentation.

**Figure 3:**
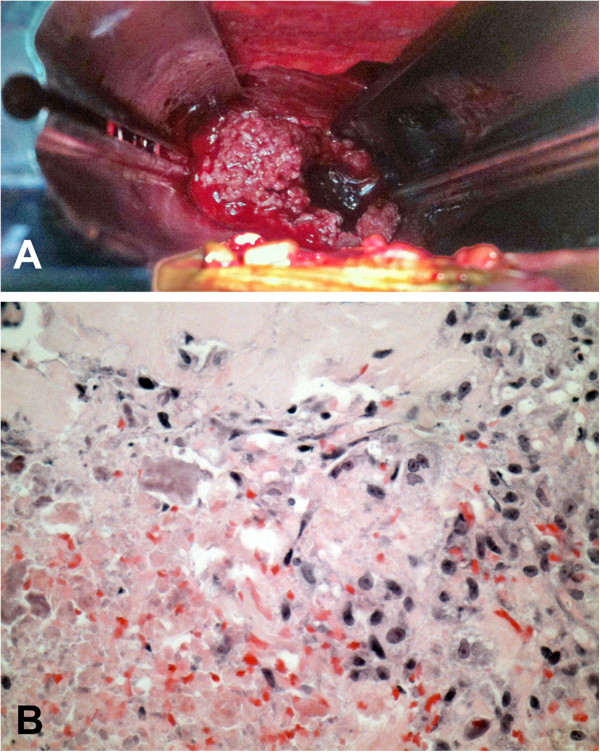
**Intra-operative photograph and histological section showing necrotic tissue and bony debris consistent with Charcot changes. (A)** Intra-operative photograph at time of anterior procedure showing necrotic tissue and bony debris in place of the L4 vertebral body. **(B)** Histological section showing necrotic tissue at bottom left of slide and macrophage and fibroblastic reaction top right, consistent with Charcot changes (magnification ×400, hematoxylin and eosin stain).

A staged combined surgical approach was undertaken in order to obtain circumferential arthrodesis. The first surgery involved a posterior approach and extension of the previous fusion from L3 to S1 with pedicle screws and rods (EXPEDIUM® Spine System, DePuy Spine, Raynham, MA, USA; Figure 2). These were connected to the previous spinal instrumentation using rod connectors and cross-links. The second surgery was performed after 1 week and involved a direct lateral trans-psoas retroperitoneal approach to L4, where extensive destruction with bony debris was observed. L4 corpectomy and anterior reconstruction using an expandable titanium cage stabilized with vertebral body staples and screws in L3 and L5 held with a 6.25mm diameter rod (LEGACY™ Spinal System, Medtronic Sofamor Danek, Memphis, TN, USA) was performed (Figure 2). Histopathology of the corpectomy specimen showed necrotic tissue associated with nonspecific chronic inflammation, fibrosis and granulation tissue with no evidence of infection or malignancy (Figure 3). Culture results were negative for bacteria, acid-fast bacilli, *Brucella*, and fungi.

**Figure 4:**
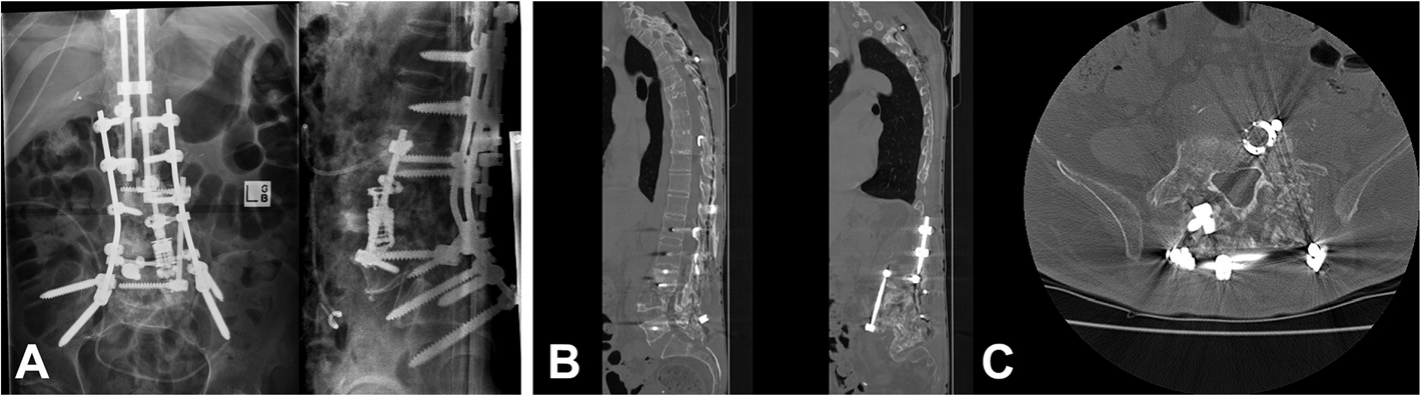
**Plain radiographs and computed tomography scan showing revision and extension of posterior instrumentation to the ilium. (A)** Anteroposterior and lateral radiographs showing revision and extension of posterior instrumentation to the ilium. **(B)** and **(C)** Sagittal and axial computed tomography scan taken after 6 weeks of immobilization following the revision surgery showing consolidation of the bone graft.

The patient was mobilized postoperatively but after 10 days unfortunately experienced recurrence of the clicking when sitting forward, associated with recurrence of intermittent sweats. Repeat X-rays showed disengagement and subluxation of the anterior cage, suggesting persisting instability of the Charcot spine at the operated level (Figure 4). He thus subsequently underwent a third surgery which was performed posteriorly, revealing severe loosening of the L5 and S1 screws bilaterally and involving revision and extension of the posterior instrumentation to his ilium with screws and cobalt-chrome rods (Expedium). Morcellized femoral head allograft and recombinant human bone morphogenetic protein-2 (INFUSE®, Medtronic Sofamor Danek, Memphis, TN, USA) were added to the decorticated bone. He was then immobilized in bed for 6 weeks. After this time, a repeat CT scan of his spine showed posterolateral spinal fusion consolidation of the bone graft and fusion mass from T4 to his ilium (Figure 4), he was allowed to mobilize and did not have recurrence of his symptoms.

**Figure 5:**
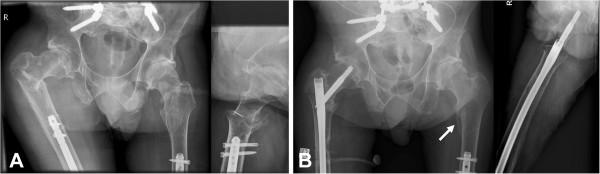
**Plain radiographs taken 10 weeks after the final spinal surgery showing intertrochanteric right neck of femur fracture distal to the iliac fixation and subsequent intramedullary fixation. (A)** Radiographs taken 10 weeks after the final spinal surgery showing intertrochanteric right neck of femur fracture distal to the iliac fixation. **(B)** Radiographs taken 6 weeks post-intramedullary fixation showing stable fixation of the right femoral fracture. A stress response with periosteal new bone formation in the left subtrochanteric region is apparent (arrow).

At 10 weeks after his final spinal surgery and 4 weeks after recommencing mobilization, he heard a sudden crack in his right hip region when bending forward in his wheelchair. This was associated with deformity and swelling around the hip region and an immediately floppy leg. X-rays showed a comminuted intertrochanteric right femoral neck fracture, between the previous spinopelvic fixation and the retrograde femoral nail (Figure 5). The spinopelvic instrumentation was unchanged. He underwent further surgery involving removal of the previous retrograde femoral nail, reduction of the intertrochanteric femoral neck fracture and fixation with an antegrade femoral intramedullary device (Long Gamma® Locking Nail, Stryker Orthopaedics, Mahwah, NJ, USA). He made an uneventful recovery and was able to mobilize as tolerated. Of interest, radiographs taken at 6 weeks postoperatively showed a stress response of the bone on the contralateral (left) side, with periosteal bone formation in the subtrochanteric region (Figure 5).

## Discussion

The development of Charcot spine arthropathy is a rare occurrence but should be considered in every active patient with paraplegia presenting with back pain and/or deformity distal to the sensory level, change in neurological function and symptoms of autonomic dysreflexia [4]. Surgery is indicated after failure of non-operative management for pain, neurological deterioration, progressive deformity, impairment of sitting balance and autonomic dysreflexia. This case highlights the many difficult issues faced in surgery for Charcot spine, which may involve dual surgical approaches, revising previous spinal instrumentation, extending an already long spinal fusion, and correction of deformity in the presence of suboptimal bone quality. Consequently, there is a high risk of associated complications, particularly with failure of instrumentation and adjacent distal level failure. In this case, the distal L5 and S1 screws implanted in the first procedure failed bilaterally by loosening within the bone, despite anterior surgery, reconstruction and stabilization. It is uncertain whether or not this might have been prevented with the use of polymethylmethacrylate augmentation of the pedicle screws. Nevertheless, revision surgery and extension of the long multisegmental posterior thoracolumbar fusions to the ilium with two screws bilaterally was performed.

Long rigid multisegmental fusions into the lumbosacral spine have been shown to significantly increase the stress and motion at more distal segments, predisposing these segments to degeneration and premature failure in the form of insufficiency fracture, especially in patients with osteoporosis [10,11]. Sacral insufficiency fractures are potential complications following lumbosacral fusion. Extended spinopelvic fixation to the ilium may improve fusion rates and provide a significant biomechanical advantage to secure distal fixation of long instrumented fusions to the pelvis by protecting the S1 screws from pullout and decreasing lumbosacral motion [13]. However, iliac wing screws have complication rates of up to 50%, including screw prominence and local irritation, screw loosening and breakage, and infection [14,15]. We report a case of intertrochanteric femoral neck insufficiency fracture following spinopelvic fixation. It is likely that rigid multilevel instrumentation of the spine and the pelvis transferred stress to the adjacent distal joint, in this case the hip joint, which failed under increased strains of torsional loading.

## Conclusions

Spinal Charcot’s arthropathy is a rare condition that may occur in patients with post-traumatic spinal cord injury. Although associated with high risk of complications, circumferential instrumented fusion in Charcot spine can restore spinal stability. Insufficiency fractures of the proximal femur are possible complications of long spinopelvic fusions. The biomechanical effects of long fusions and instrumentation to the ilium need to be further studied. Femoral neck insufficiency fractures are possible complications of spinal fusions to the pelvis.

## Consent

Written informed consent was obtained from the patient for publication of this case report and accompanying images. A copy of the written consent is available for review by the Editor-in-Chief of this journal.

## Competing interests

The authors declare that they have no competing interests.

## Authors’ contributions

Both authors treated the patient and were major contributors in writing the manuscript. Both authors read and approved the final manuscript.
